# DHA Serum Levels Were Significantly Higher in Celiac Disease Patients Compared to Healthy Controls and Were Unrelated to Depression

**DOI:** 10.1371/journal.pone.0097778

**Published:** 2014-05-19

**Authors:** Nathalie J. M. van Hees, Erik J. Giltay, Johanna M. Geleijnse, Nadine Janssen, Willem van der Does

**Affiliations:** 1 Institute of Psychology, Leiden University, Leiden, The Netherlands; 2 Department of Psychiatry, Leiden University Medical Center, Leiden, The Netherlands; 3 Division of Human Nutrition, Wageningen University, Wageningen, The Netherlands; 4 Leiden Institute of Brain and Cognition, Leiden, The Netherlands; Chiba University Center for Forensic Mental Health, Japan

## Abstract

**Objectives:**

Celiac disease (CD), a genetically predisposed intolerance for gluten, is associated with an increased risk of major depressive disorder (MDD). We investigated whether dietary intake and serum levels of the essential n-3 polyunsaturated fatty acids (PUFA) eicosapentaenoic acid (EPA) and docosahexanoic acid (DHA) found in fatty fish play a role in this association.

**Methods:**

Cross-sectional study in 71 adult CD patients and 31 healthy volunteers, matched on age, gender and level of education, who were not using n-3 PUFA supplements. Dietary intake, as assessed using a 203-item food frequency questionnaire, and serum levels of EPA and DHA were compared in analyses of covariance, adjusting for potential confounders. Serum PUFA were determined using gas chromatography.

**Results:**

Mean serum DHA was significantly higher in CD patients (1.72 mass%) than controls (1.28 mass%) after multivariable adjustment (mean diff. 0.45 mass%; 95% CI: 0.22–0.68; *p* = 0.001). The mean intake of EPA plus DHA did not differ between CD patients and controls after multivariable adjustment (0.15 and 0.22 g/d, respectively; *p* = 0.10). There were no significant differences in intake or serum levels of EPA and DHA between any of the CD patient groups (never depressed, current MDD, minor/partially remitted MDD, remitted MDD) and controls.

**Conclusions:**

Patients on a long term gluten-free diet had similar intakes of EPA plus DHA compared to controls. Contrary to expectations, DHA serum levels were significantly higher in CD patients compared to healthy controls and were unrelated to MDD status.

## Introduction

Celiac disease (CD) is a genetically predisposed intolerance for gluten that affects approximately 1 in 160 people [1]. CD is caused by an inappropriate enhanced immune response of the T-lymphocytes of the small intestines to gluten peptides. This results in intestinal malabsorption, atrophy of the intestinal villi and chronic inflammation of the jejunal mucosa of the small intestine. There is currently no cure for CD, but a gluten-free diet improves the histopathology as well as symptoms like weight loss, steatorrhea, diarrhea, abdominal distension, and pain [2]. Besides these intestinal problems, CD is associated with an almost doubled prevalence of major depressive disorder (MDD) [3]–[9]. Its prevalence rate remains high when a gluten-free diet is initiated [10], [11], and may even increase after initiation of the gluten-free diet [12]–[15].

Although the burden of having a chronic disease might be sufficient to cause MDD in some patients nutrient deficiencies due to malabsorption and the mandatory restrictive diet may also contribute. Treated CD patients often obtain restoration of the function and structure of their atrophied intestinal villi which should correct their malabsorption problems [16], but the strict gluten-free diet may induce nutrient deficiencies in itself. The gluten-free diet has been found to be low in micronutrients and fatty acids like iron, calcium, B vitamins, alpha-linolenic acid and arachidonic acid [17]–[19], and CD patients may avoid high fat meals (including fatty fish) that induces steatorrhea and other intestinal problems. Eicosapentaenoic acid (EPA, 20∶5n-3), docosahexaenoic acid (DHA, 22∶6n-3) and alpha-linolenic acid (ALA, 18∶3n-3) are essential long-chain n-3 polyunsaturated fatty acids (PUFA) that are important components of the human diet. EPA and DHA are found in fatty fish, while ALA is found in green vegetables, nuts (e.g. walnuts), and vegetable oils (e.g. canola and soybean oils). There is only a minor pathway of biosynthesis from the precursor ALA to EPA and DHA with an approximately 10–15% efficiency [20], [21], and vegetarians and persons who do not eat fish may depend on this metabolic pathway for their n-3 PUFA. EPA and DHA concentrations in plasma phospholipid have been found to largely reflect dietary intakes of these fatty acids. DHA comprises about 30% of the fatty tissue in the central nervous system [22] and is a precursor to the signaling eicosanoid molecules prostaglandins and leukotrines involved in the regulation of inflammation and microvascular control. EPA and DHA are considered to have anti-inflammatory effects in the human body [23].

There is evidence that an increased dietary intake of DHA and EPA, and possibly ALA, may lower the risk of MDD [24]–[26]. Also, circulating levels of n-3 PUFA (or their ratio to n-6 unsaturated fatty acids) have been inversely associated with MDD [27], [28] and depressive symptoms [29]. Randomized trials with n-3 PUFA supplementation studies have shown mixed results [30]–[34].

CD is associated with a higher prevalence of MDD [3]–[9]. Several studies have found that the daily intake of total fat is significantly higher in CD patients. Furthermore, CD patients’ total energy intake is significantly lower than that of healthy controls [35]–[37] but no previous study has analyzed the intake of n-3 PUFA in CD patients or its relationship with MDD. Several paediatric studies suggest that the lipid profile is different in CD patients than in healthy controls, but most did not focus on n-3 [38]–[40]. A small paediatric study found no significant difference in total serum n-3 fatty acids among 7 patients with active CD, 6 patients in remission and 11 controls, however arachidonic acid to DHA ratio in patients in remission was significantly higher than in controls [41]. In adults, DHA and EPA serum levels were significantly lower than in controls at time of diagnosis and after one year of gluten-free diet treatment [42].

In summary, n-3 fatty acid intake and blood levels seem to be associated with MDD. Some studies suggest an association between CD and circulating n-3 fatty acid levels, but studies are small and mainly done in children. N-3 fatty acid intake has not previously been measured in CD patients. Our aim was to investigate whether dietary intake and serum levels of the essential n-3 polyunsaturated fatty acids (PUFA) eicosapentaenoic acid (EPA) and docosahexanoic acid (DHA), play a role in the association between CD and depression. We hypothesized that the gluten-free diet may cause low EPA and DHA intake and serum levels, resulting in an increased risk of MDD in patients with CD. Therefore, we compared intake and serum levels of EPA and DHA among groups of CD patients with and without MDD, and compared dietary intake of EPA and DHA in CD patients with healthy controls matched on age, gender and level of education.

## Methods

### Participants

**Figure 1:**
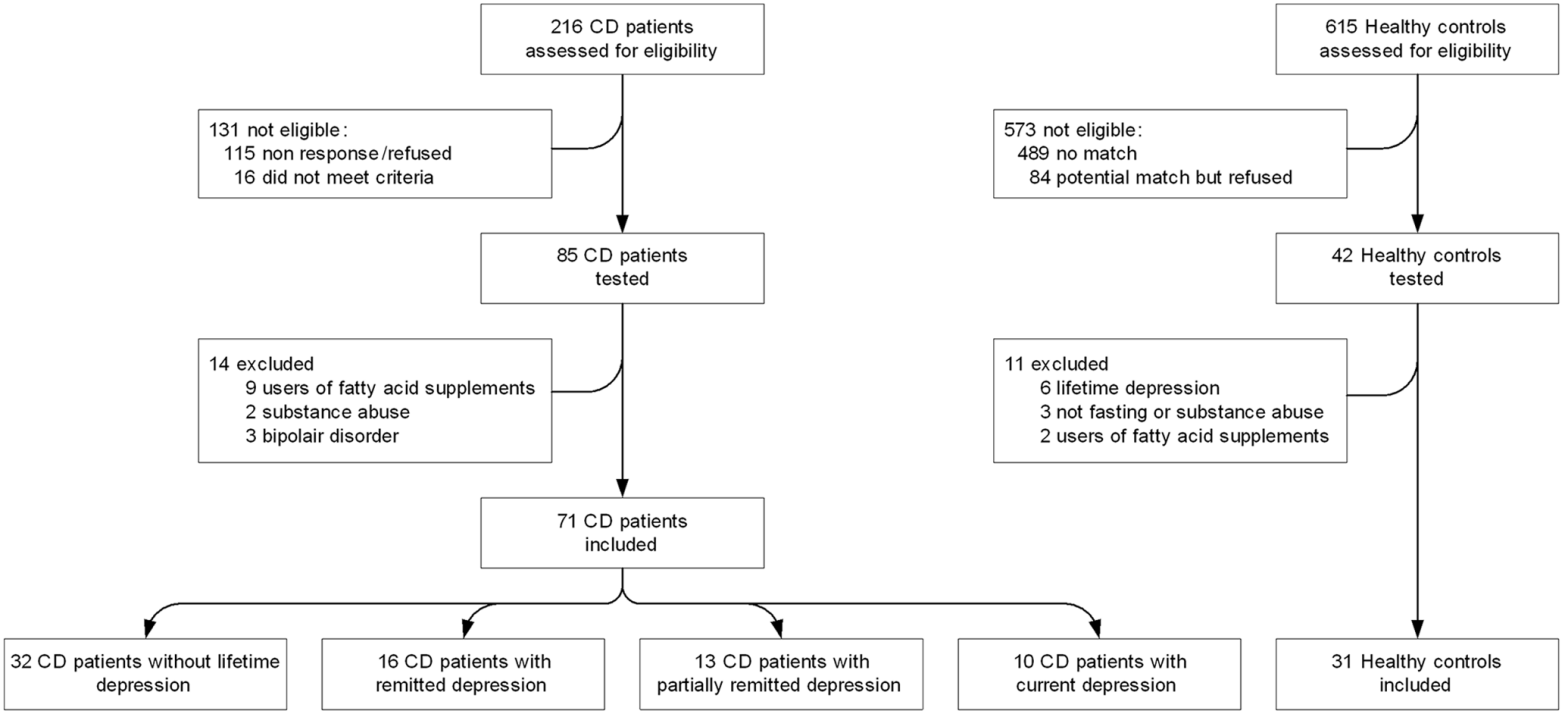
Flow chart of participants in the study.

A cohort of CD patients was recruited from the 2,265 participants (age 18–93 y) of a previous survey study [9] performed among adult members of the Dutch Celiac Association (NCV), as shown in a flow chart (Figure 1).

216 CD patients in the regions Leiden and Amsterdam were contacted and screened for assignment to the never depressed, remitted depressed and currently depressed CD patient study conditions. Participants with self-reported depressive symptoms were oversampled in order to obtain equal group sizes. Healthy, never-depressed controls were recruited from the 1,295 participants of another study that aimed to gather reference data from the general population (‘Normquest’ study) [43]. Eligible healthy participants in the Leiden region (N = 615) were pre-screened for mood disorders and were matched for age, gender and level of education. Between October 2010 and April 2011, 85 CD patients and 42 controls took part. Written confirmation of CD diagnosis was requested from the treating specialists and was obtained for all but 8 (11.2%) participants. Participants were excluded if they were younger than 18, had ulcerative colitis, Crohn’s disease, current chemotherapy or conditions which would make the testing session unreliable or impossible such as severe psychosis, mental retardation, blindness or deafness. CD participants were excluded if they were on the gluten-free diet less than 2 years or had self-reported low adherence to the gluten-free diet. Healthy controls where excluded if they had celiac disease, shared a household or had a 1^st^ degree family relation with a CD patient or had any mood disorder diagnosis on MINI-plus interview [43]. For the current analyses the following subjects were excluded (Figure 1): those with missing data on any main variables (n = 0), bipolar disorder (n = 3), current alcohol abuse (n = 3), current drug abuse (n = 1), not fasting on morning of testing (n = 1), healthy control with lifetime diagnosis on repeated MINI-plus interview of any mood disorder (n = 6), use of fatty acid (i.e., n-3 PUFA) food supplements (n = 11).

### Procedure

In accordance with the declaration of Helsinki, this study was reviewed and approved by the Medical Ethics Committee of the Leiden University Medical Centre and all participants provided written informed consent before the start of data collection. Participants had the capacity to consent, as assessed during screening, and there was no surrogate consent procedure. The interview and the blood collection was performed at the Leiden University Medical Center or at a participating general practitioners office in Amsterdam. Six participants were tested at home due to their advanced age or disability. Participants were sent the study information and instructions, the food frequency questionnaire, the Celiac Disease Adherence Test, a Lifestyle and Health questionnaire, and an informed consent form two weeks before the day of testing. Participants were fasting and refrained from smoking in the hour prior to blood sampling. The testing day started with the physical examination and blood collection, after which participants consumed a light breakfast.

### Instruments

#### Psychiatric diagnoses

The Dutch version [44] of the complete Mini International Neuropsychiatric Interview Plus 5.0.0-R (MINI-Plus) was administered [45]. The MINI is a structured clinical diagnostic interview of current and lifetime Axis-I disorders according to the criteria of the Diagnostic and Statistical Manual of Mental Disorders – Fourth Edition (DSM-IV) [46]. A minimal modification was made to the criteria for scoring ‘MDD partially in remission’, using the criteria suggested by Rush et al. [47]. Participants who had never experienced a mood disorder were placed in the ‘never’ group. Participants with dysthymia were placed in the MDD groups (n = 1 in the current MDD, and n = 2 remitted MDD group). Participants who were currently suffering from an episode of MDD or dysthymia were placed in the ‘current’ group. Participants who recently had an episode of MDD or dysthymia but who now had subclinical symptoms were placed in the ‘partially remitted’ group. Participants who had suffered from MDD, and currently experienced an absence of both sad mood and reduced interest and no more than three of the remaining seven symptoms of MDD for three or more weeks were placed in the ‘remitted’ group. All participants were tested and interviewed by two interviewers (from five interviewers in total). At the end of each session both raters tried to reach agreement on all diagnoses. When agreement could not be reached, an intervision meeting was scheduled with the full research team during which consensus was reached.

#### Food frequency questionnaire

We used an updated version of a validated semi-quantitative food frequency questionnaire that was previously used in epidemiological studies in the Netherlands (45,47,48) The questionnaire covers the 1-month intake of 203 food items and beverages. Information on use of food supplements is also obtained. The questionnaire was sent to the participants and filled in at home. A data check for completeness was performed during the visit to the study center. The food frequency questionnaire was not designed to take the gluten-free diet into account, and therefore additional questions on the ingredients of the gluten-free food products were included. Also, we asked participants to provide the packaging and labels of the gluten-free products that they had used in the past month. Nutrient intake was calculated using the Dutch Food Composition Table (‘Nederlands Voedingsstoffenbestand’; NEVO, 2006), which was extended for gluten free products by a dietician [48].

#### Other variables

Body weight (kg) and height (cm) were measured and body mass index (kg/m^2^) was computed. Blood pressure was measured twice after a 5-minute rest, once lying down before breakfast and once sitting up after having breakfast. Physical activity was assessed using the Physical Activity Scale for the Elderly (PASE) [49], to estimate ‘metabolic equivalents of task’ (MET) minutes. Smoking behavior, alcohol consumption, and self-reported medication use was assessed using questionnaires. Furthermore the nature and method of CD diagnosis were assessed, as well as current and lifetime medical disorders.

#### Blood sampling and other measures

Fasting venous blood samples were obtained on ice, centrifuged and serum was kept at −80°C within 3 hours after collection. The fatty acids (omega-3, omega-6 and omega-6:omega-3 ratio) from total lipids and high-sensitivity C-reactive protein (hsCRP) were assessed. hsCRP concentrations (mg/L) were measured using nephelometry. Fasting serum fatty acids were determined as percentage of total fatty acids by a slightly modified gas chromatographic procedure as described by Lepage and Roy [50]. To 100 µL of serum 15 µL of a 1.0 mg/ml solution of heptadecanoic acid in chloroform/methanol (1∶1; v/v) was added as an internal standard and subsequently 2.0 ml of methanol/benzene (4∶1; v/v). Then 200 µL of acetylchloride was added slowly and derivatization was performed for 1 h at 100°C. After adding 5 ml of a 6% (w/v) potassium carbonate solution in water and cooling of the mixture it was centrifuged and as much as possible of the benzene upper layer was isolated. This was dried under a gentle nitrogen flow and the residue was taken up in 50 µL of hexane. Finally 1 µL of this sample was injected using split-injection (1∶20) on a Trace/Focus gaschromatograph (Interscience, Breda, The Netherlands) using a 30 m capillary BPX-70 column (SGE, Ringwood, Australia) and fatty acids were quantified by calculating the peak area ratios of the fatty acids and the internal standard.

#### Assessment of adherence to gluten-free diet and CD diagnosis

The current level of adherence to the gluten-free diet was assessed with a single self-report question [9] as well as with the Celiac Disease Adherence Test (CDAT) [51]. In addition a series of questions was asked to assess diet history (duration, age at onset, and any diet interruptions).

### Statistical Analysis

Group differences were analyzed using chi-squared (χ^2^) tests for categorical variables and analysis of variance (ANOVA) for continuous variables. Analysis of covariance (ANCOVA) was used to adjust for age, gender, level of education, BMI, smoking, alcohol use and statin use in model 1. To assess the potential mediation by differences in dietary intake, we additionally adjusted for daily intake of EPA and DHA when analyzing serum levels of n-3 PUFA in model 2. Odds ratios were calculated using logistic regression analysis assessing the risk of MDD according to EPA and DHA intake and serum EPA and DHA levels. Statistical significance was inferred at a two-sided p<0.05. Analyses were done with SPSS software (Version 19.0. Armonk, NY: IBM Corp).

## Results

### Participant Characteristics

No participant had missing data on the main study variables. Patients with CD were on average 54 years old (range 20–86 years) and 76% was female (Table 1). Healthy controls were on average 51 years old (range 22–66 years), 65% was female. Both groups had an above average education level. The CD group (n = 71) comprised 46 participants (65%) who had one or more (up to 4) current psychiatric diagnoses, mainly anxiety disorders. CD patients were engaged in physical activity 60 MET hours per month less than healthy controls (*p* = 0.03). On average, CD patients were maintaining a gluten-free diet for an uninterrupted period of 15.1 years (SD = 11.5), ranging between 2.6 and 52 years. Length of current gluten-free diet did not differ significantly among the 4 depression groups of CD patients. Self-reported diet adherence in our sample could be categorized as ‘very strict’ in 74% of participants, ‘strict’ in 24%, and only 1% for ‘moderately well’ to ‘poor’. Diet adherence according to Celiac Disease Adherence Test results was categorized as ‘excellent’ or ‘very good’ in 63% of participants, ‘very good’ to ‘fair’ in 26% and ‘fair’ to ‘poor’ in 11%.

**Table 1:** Socio-demographic and medical characteristics in celiac disease patients and matched controls.

	Controls (n = 31)	Celiac disease (n = 71)	*P*-value*
Age (years)	51.1±13.3	53.9±18.7	0.45
Gender			
- Male	11 (35%)	17 (24%)	0.23
- Female	20 (65%)	54 (76%)	
Level of education			
- Low	7 (22.6%)	18 (25.4%)	0.52
- Intermediate	6 (19.4%)	20 (28.2%)	
- High	18 (58.1%)	33 (46.5%)	
Body mass index (kg/m^2^)	24.9±3.7	24.6±4.0	0.72
Blood pressure			
- Systolic (mmHg)	120.5±20.3	125.7±21.6	0.25
- Diastolic (mmHg)	72.6±10.5	69.2±10.8	0.14
Statin use	6 (19.4%)	7 (9.9%)	0.19
Current smoker	7 (22.6%)	7 (9.9%)	0.09
Alcohol intake			
- No	10 (32.3%)	31 (43.7%)	0.28
- 1–2 glasses/d	14 (45.2%)	32 (45.1%)	
- ≥2 glasses/d	7 (22.6%)	8 (11.3%)	
Number comorbid diseases	1.0 (0.0–2.0)	2.0 (1.0–3.0)	0.02
hsCRP (mg/L)	0.94 (0.50–2.31)	0.95 (0.31–2.13)	0.11
Physical activity (MET hours/week)	44.5±35.1	30.6±25.1	0.03

Data are presented as n (%), mean (± SD) or median (Q_1_–Q_3_), when appropriate. ALA denotes alpha-linolenic acid; DHA, docosahexaenoic acid; EPA, Eicosapentaenoic acid; hsCRP, High-sensitivity C-reactive protein; MET, metabolic equivalents of task.

*P-values by chi-squared test for categorical variables and by ANOVA for continuous variables.

### Dietary Intake of Fat and Fatty Acids

Nutrient value tables specifically for the gluten-free diet were used, which did not affect the estimations of EPA plus DHA intake. Table 2 shows a no significant difference in overall intake of fat and fatty acids nor were there significant differences after controlling for covariates. The intake of EPA plus DHA was not significantly different in CD patients compared to controls (mean 0.17 and 0.21 g/d, respectively; *F*(1,100) = 1.06*; p* = 0.31) nor after controlling for covariates (mean 0.15 and 0.22 g/d, respectively; mean diff. 0.073 g/d; 95% CI: −0.015–0.161; *p* = 0.10). The MDD groups did not differ from controls on this variable either. The intakes of total energy, total fat, unsaturated fatty acids, ALA and of EPA plus DHA did not differ significantly between CD patients and controls, nor between the CD depression groups and controls. After controlling for confounders, energy intake seemed to differ among groups, but when comparing the CD patient group as a whole to controls this difference was not significant (*p* = 0.67).

**Table 2:** Daily dietary intakes in 31 controls and 71 patients with celiac disease with and without depression.

	Controls	Patients with celiac disease				*P*-value*
Dietary intake	(n = 31)	Never MDD (n = 32)	Remitted MDD (n = 16)	Partially remitted MDD (n = 13)	Current MDD (n = 10)	
Total energy (kcal/d)	1979±98	2025±102	1798±153	1872±149	1815±165	0.64
Total fat (g/d)	72.9±4.7	73.2±5.1	66.5±6.2	76.5±7.2	70.7±7.9	0.89
Unsaturated fatty acids (g/d)	14.0±1.8	16.3±2.0	12.5±1.3	15.5±2.1	14.3±2.0	0.61
ALA (g/d)	1.03±0.08	1.23±0.16	1.09±0.12	1.08±0.11	1.05±0.15	0.78
EPA plus DHA (g/d)	0.17±0.03	0.20±0.03	0.15±0.03	0.32±0.08	0.20±0.08	0.18

Data are presented as mean (± SE), when appropriate. ALA denotes alpha-linolenic acid; DHA, docosahexaenoic acid; EPA, eicosapentaenoic acid; MDD, major depressive disorder.

*P-values by ANOVA for continuous variables; adjusted for gender, age, education, BMI, smoking, alcohol use, and statin use.

### Serum Levels of Fatty Acids

The n-6: n-3 ratio was approximately 17∶1 in both groups with no significant difference between CD patients and controls after controlling for confounders. Intake of EPA plus DHA showed a small but significant Pearson correlation to serum EPA as well as to serum DHA concentrations in our total sample (n = 102; r = 0.18, *p* = 0.08 and r = 0.27, *p* = 0.007, respectively). This indicates that fatty fish intake was indeed associated with serum DHA, more so than with serum EPA concentrations, but that it could only explain a small part of its variance. None of the tested serum PUFA levels differed significantly between CD patients (n = 71) and healthy controls (n = 31), except for DHA (table 3). DHA serum level in CD patients was significantly different from controls (mean 1.72 and 1.28 mass%, respectively; *F*(1,100) = 15,47; *p* = 0.001). After controlling for confounders the mean level of DHA in CD patients was on average 1.72 mass% versus 1.28 mass% for controls (mean diff. 0.45 mass%; 95% CI: 0.22–0.68 *p*<0.001). This difference remained significant after controlling for dietary intake of EPA and DHA as an additional covariate (mean diff. 0.39 mass%; 95% CI: 0.17–0.62 *p*<0.001), where intake of EPA and DHA contributed significantly to the regression model and explained 8% of the variance and group membership explained 12% of the variance.

**Table 3:** Serum fatty acid content in 31 controls and 71 patients with celiac disease with and without depression.

	Controls	Patients with celiac disease				*P*-value*
	(n = 31)	Never MDD (n = 32)	Remitted MDD (n = 16)	Partially remitted MDD (n = 13)	Current MDD (n = 10)	
Total fatty acids	10.01±2.06	9.47±2.14	9.86±1.52	9.86±1.76	10.27±2.56	.62
C12∶0 (Lauric A)	0.15±0.01	0.13±0.01	0.15±0.02	0.10±0.01	0.12±0.02	.16
C14∶0 (Myrisitic A)	1.26±0.06	1.19±0.06	1.22±0.11	0.97±0.08	1.21±0.16	.45
C16∶0 (Palmitic A)	25.06±0.34	24.34±0.25	24.54±0.60	24.20±0.38	24.24±0.77	.48
C16∶1. n-7 (Palmitoleic A)	2.41±0.12	2.47±0.16	2.17±0.14	1.99±0.16	2.13±0.31	.25
C18∶0 (Stearic A)	6.67±0.11	6.76±0.12	6.71±0.21	6.51±0.23	6.88±0.29	.78
C18∶1. n-9 (Oleic A)	20.95±0.50	21.30±0.44	20.63±0.73	20.73±0.70	22.00±0.89	.71
C18∶2. n-6 (LA)	28.65±0.79	27.71±0.80	28.79±1.11	29.99±1.04	28.35±1.99	.66
C18∶3. n-3 (ALA)	0.56±0.03	0.61±0.05	0.48±0.03	0.57±0.06	0.53±0.06	.35
C20∶4. n-6 (AA)	5.89±0.21	6.36±0.21	5.91±0.34	5.69±0.35	5.67±0.42	.34
C20∶5. n-3 (EPA)	0.72±0.05	0.92±0.12	0.94±0.18	0.94±0.25	0.79±0.17	.65
C22∶5. n-3 (DPA)	0.35±0.02	0.40±0.02	0.36±0.03	0.34±0.04	0.36±0.03	.22
C22∶6. n-3 (DHA)	1.28±0.06	1.65±0.09†	1.78±0.16†	1.87±0.20†	1.69±0.18†	.003**

Data are (adjusted) mean ± standard errors (SE), total fatty acids in mmol/L, individual fatty acids as a % of total fatty acids. MDD denotes major depressive disorder.

† significantly different in post-hoc tests versus the controls.

*P-values by ANOVA for continuous variables; adjusted for gender, age, education, BMI, smoking, alcohol use, and statin use.

**additionally adjusted for daily intake of EPA and DHA.

**Figure 2:**
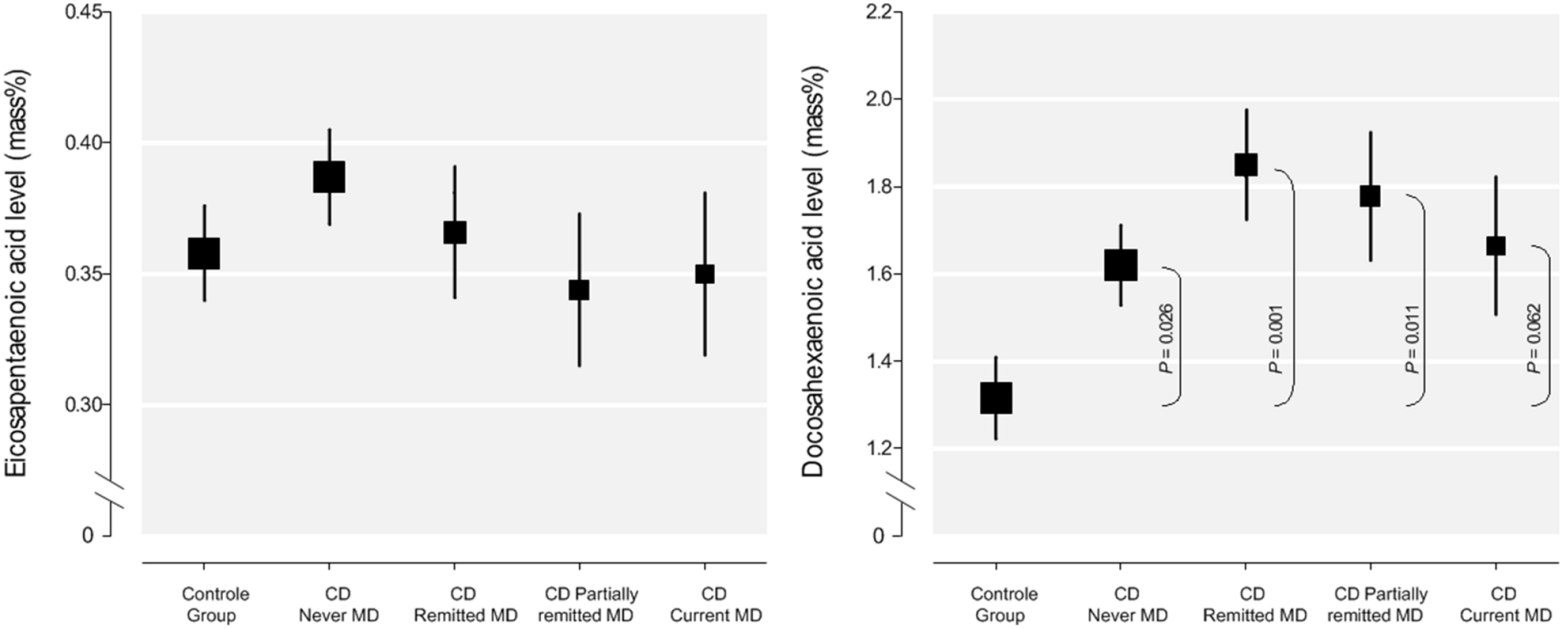
Mean standard scores (with error bars representing standard errors) for plasma levels of doxosahexaenoic acid (DHA) and eicosapentaenoic acid (EPA) in % of total fatty acids. The size of each square is proportional to the number of participants. Mean scores are adjusted for gender, age, education, BMI, smoking, alcohol use, statin use, and daily intake of EPA and DHA. *P*-values by analysis of covariance.

In post-hoc tests, we compared the mean serum levels of DHA between the 4 CD groups using ANCOVA adjusting for covariates. We did not find a significant difference in serum levels among CD depression categories (figure 2). When comparing CD patients with current and partially remitted MDD (cases) versus CD patients with no or remitted MDD (controls), continuous EPA levels were not associated with a higher risk of MDD with an odds ratio of 0.90 (95% CI: 0.44–1.85; *p* = 0.77) nor were DHA levels with an odds ratio of 1.33 (95% CI: 0.58–3.08; *p* = 0.50).

To take the influence of possible current inflammation into account we performed a sensitivity analysis where we additionally adjusted our multivariate model for log-transformed hsCRP levels, which did not alter the results for serum EPA and DHA levels (data not shown). In another sensitivity analysis, we excluded the 6 CD participants for whom the potential confirmed CD diagnosis could not be retrieved. Again this did not alter our results for serum EPA and DHA levels (data not shown). We investigated the relationship between gluten-free diet characteristics and DHA serum levels and DHA intake. Adherence to the gluten-free diet as measured by the Celiac Disease Adherence Test did not predict DHA serum levels (*p* = 0.27), nor DHA intake (*p* = 0.72). Length of gluten-free diet did not predict DHA serum levels (*p* = 0.77) or DHA intake *(p* = 0.70).

## Discussion

Our study showed that treated CD patients had a higher serum DHA level than healthy controls. This does not seem to reflect an increased intake of EPA and DHA by CD patients on a gluten-free diet, as fish fatty acid intake did not differ significantly among these groups. We found no association between dietary intake or serum levels of EPA or DHA and MDD status within the group of CD patients, and therefore the differences in DHA levels could not explain the differences in occurrence of MDD.

There is evidence that an increased dietary intake of DHA and EPA, and possibly ALA, may lower the risk of MDD [24]–[26]. Also, circulating levels of n-3 PUFA (or their ratio to n-6 unsaturated fatty acids) have been inversely associated with MDD [27], [28] and depressive symptoms [29]. Randomized trials with n-3 PUFA supplementation studies have shown mixed results [30]–[34]. Some reviews have found lower EPA and DHA plasma levels in depressed patients [30] and a small but significant benefit of EPA and DHA supplementation in MDD patients [31]. Other reviews found no effect of EPA and DHA supplementation on MDD [32] and no beneficial effect of EPA and DHA supplementation on mood in women with perinatal depressive symptoms [33] or subjects not suffering from current MDD [34].

We found increased serum DHA in our CD patients, but no difference in serum EPA, compared to controls which is inconsistent with the literature. Although the literature on n-3 fatty acid metabolism in patients with CD, or chronic diseases in general, is very limited, data suggest that severe malabsorption and chronic gastrointestinal disorder is associated with essential fatty acid deficiencies; in particular linoleic acid and DHA [52], [53]. Some studies even propose permanent changes in fatty acid metabolism [52]–[55]. Studies on n-3 fatty acid plasma levels and fatty acid profiles in CD patients have shown mixed results, in particular between studies done in paediatric [38], [39], [41] and adult samples [42], [56]. All studies however show unfavorable differences in CD patients’ fatty acid profiles when comparing them to healthy groups. For example, a study assessing recently diagnosed adult CD patients found that patients’ DHA, EPA and arachidonic acid serum levels increased after a one-year strict gluten-free diet but stayed significantly lower than those of controls. Serum arachidonic acid and DHA levels improved most. As the authors propose in their discussion, essential fatty acid concentration may continue to increase after following a gluten-free diet for a longer period of time. [42]. Studies into CD or the gluten-free diet are difficult to compare however since differences in stages of disease activity of CD, different length of the gluten-free diet and level of adherence to the gluten-free diet need to be taken into account. For example, paediatric patients with active CD had significant signs of essential fatty acid deficiency, but when these patients were in remission and on a gluten-free diet for one year or longer, their DHA levels were not significantly lower than those of controls [41]. In contrast, in our study in CD patients on a long term gluten-free diet (mean 15 years) serum DHA levels were significantly higher than healthy controls. As some authors have previously suggested permanent changes in fatty acid metabolism in chronically ill samples [52]–[55], we speculate that such a change may have occurred in our sample of CD patients in remission. Possibly through sustained activation of counterbalancing (e.g. anti-inflammatory) processes that help to restore homeostasis, which might have involved the formation of DHA [57]–[60]. After antigen exposure is eliminated, chronic inflammation might slowly be reduced by anti-inflammatory mechanisms including the increased production of DHA. We hypothesize that the prolonged activation of this process might have resulted in a permanent up-regulation of DHA formation.

An alternative explanation is a change in PUFA intake due to the exclusive nature of the gluten-free diet. Our questionnaire data did however not reveal a significantly different dietary intake of PUFA or total fat between patients and controls. It is therefore less likely that our finding of an elevated DHA serum level is attributable to differences in DHA intake as a result of the gluten-free diet. We also found that using nutrient tables designed for the gluten-free diet did not really alter the outcome of the FFQ on variables involving fatty acids. This leads us to conclude that the gluten-free diet does not really have an impact on main dietary sources of fatty acids. Contrary to our findings most previous studies found an increased intake of total fat in treated CD patients [61]–[63]. But one study found equal fat intake when comparing female participants only [64]. The lack of difference in energy intake between treated CD patients and healthy controls we found in our study is in line with earlier findings [36], [61], [62], [64]. Some other studies however found a significantly lower intake of energy [65] or higher intake of energy [63], [64]. Our findings point to a normalization of fat and energy intake in CD patients who have been living with the gluten-free diet for a long time. Whether this finding is generalizable to samples from other populations remains to be seen.

EPA and DHA supplementation in chronic inflammatory diseases such as rheumatoid arthritis, Crohn’s disease and multiple sclerosis may have beneficial effects on disease activity [66], but it is unclear whether this also applies to CD. Because serum EPA and DHA in our CD patients with depressive symptoms were similar to levels in non-depressed CD patients, we consider a beneficial effect of supplemental n-3 PUFA intake less likely. However, well-designed randomized trials in CD patients with MDD are warranted to definitely refute this hypothesis.

There are some limitations that need to be addressed. First, our sample was relatively small. Second, as we did not include patients with high dietary gluten exposure, our findings cannot be extrapolated to CD patients not on a gluten-free diet or with poor gluten-free diet adherence. However, previous research did not show a relationship between level of diet adherence and psychopathology [9], [67] nor is a relation likely between very small dietary transgressions and medical symptoms [68]. Third, both interviewers and participants were aware of the purpose of the study giving room to bias in the assessment of MDD. This possible bias was addressed to some extent by having every diagnostic interview observed by a second rater and discussed in intervision.

### Clinical Implications and Future Research

DHA serum levels were significantly higher in CD patients on a long term strict gluten-free diet and presumed in remission than in healthy controls, which may reflect alterations in fatty acid metabolism in response to the prolonged period of intestinal inflammation. Within the group of CD patients, we found no association between dietary or serum EPA plus DHA and depression status. Therefore, our findings do not support the hypothesis that supplementation of n-3 PUFA in CD patients after the first years of gluten-free diet is warranted to reduce the risk of MDD. Nevertheless, our findings warrant confirmation by other studies, preferably randomized controlled trials.
